# Internet Addiction and Its Associated Factors among Undergraduate Students in Kathmandu, Nepal

**DOI:** 10.1155/2023/8782527

**Published:** 2023-04-08

**Authors:** Sonu Acharya, Laxmi Adhikari, Santosh Khadka, Shishir Paudel, Maheshor Kaphle

**Affiliations:** Department of Public Health, CiST College, Kathmandu 44600, Nepal

## Abstract

**Background:**

Internet has penetrated all processes of life and has become an unavoidable part of people's daily life. This widespread use of the Internet has resulted in significant concerns with regard to problematic Internet behaviours and related conditions. The aim of the study was to find out the prevalence of Internet addiction and its associated factors among undergraduate students in Kathmandu.

**Materials and Methods:**

We included all together 344 undergraduate students from different colleges affiliated to Pokhara University for this cross-sectional study. We used self-administered questionnaire consisting of the Internet Addiction Test scale to assess the Internet addiction. We coded the data, entered it in Epi-Data 3.1 and transferred to IBM SPSS 25 for analysis. We applied bivariate and multivariate logistic regression analysis to identify factors associated with Internet addiction, and *p* value <0.05 was considered as statistically significantt.

**Results:**

The prevalence of Internet addiction was found to be 29.90% (95% CI: 25.0–34.9). In the chi-squared test relationship with parents, parental control over the Internet use, perceived feeling of loneliness, and time spent on internet per day were found to be statistically associated (*p* < 0.05) with Internet addiction.

**Conclusion:**

This study revealed nearly one-third of the Internet addiction among undergraduate students. Relationship with parents, parental control over the internet use, perceived loneliness feelings, and time spent on internet per day were significantly associated with Internet addiction along other factors. Therefore, it is important to raise awareness among young generation, parents, and teachers towards risk of Internet addiction.

## 1. Introduction

Internet is considered to be the most widely used media in the world, and it varies from other types of media. It has penetrated all process of life [1]. Internet invention has changed the various aspects of individual life such as in the way individuals entertain themselves and interact with each other, with the infinite social networking sites [2]. The number of Internet users has grown exponentially in the world; the number of active internet users in the world far exceeds 4 billion people [3].

Globally, there are 5.32 billion (67% of global population) mobile users, 5.00 billion people (63% of global population) using the internet and there are 4.65 billion (58.7% of global population) social media users around the world in April 2022. The annual growth rate of internet user's is 4.1% (i.e., 196 million people). Average duration of the internet use is 6 hours and 57 minutes per day on connected activities. Younger people tend to spend more time online than older generations do, with young women spending the greatest amount of time using the internet [4].

Worldwide, the prevalence of internet addiction has been estimated at 6%, considering that only about 39% of the world population has internet access. There seems to be a significant variation in rates of internet addiction between countries [5]. The prevalence of severe problematic Internet use (PIU)/Internet addiction ranged from 0 to 47.4%, whereas the prevalence of Internet overuse/possible Internet addiction ranged from 7.4% to 46.4% among students from Southeast Asia [6]. The extreme use of internet showed the addictive behaviour of the internet use [7]. Physical impairments in the form of insomnia (26.8%), daytime sleepiness (20%), and eye strain (19%) were reported among users [6]. Internet users in Nepal increased by 822 thousand (+7.7 percent) between 2021 and 2022 [8].

This widespread use of the Internet has resulted in the significant concerns with regards to problematic Internet behaviours and related conditions [9]. Internet addiction (IA) is the lack of ability to control the Internet use and involvement leading to progressive loss of control. With negative social effects, Internet addicts use the web as a social and communication tool, once they experience higher levels of pleasure and satisfaction when online than in real life [10]. It reduces social interaction to family, friends, and community leading to mental health problems such as depression, anxiety, and other psychological problems [11, 12]. Internet addiction symptoms express the users' urge to continue being connected despite the desire to stop, experiencing unpleasant emotions when they do not succeed, sleep disturbance, angry or agitated reaction when forced to disconnect, and loosing track of time while online [13]. People having a high level of anxiety use social media more which tentatively increase the negative emotions and leads to mental consequences [14, 15].

Internet is being an unavoidable part of day-to-day life because the usage of the internet has been growing explosively worldwide. With the increase in the dependence of internet, people are gradually getting addicted towards it. There are only limited studies conducted in Nepal which primarily focus on adolescents and medical students. This study focuses on undergraduate level students as this age group mostly uses internet for different purposes. They are particularly vulnerable to problematic internet use due to several factors such as the psychological and developmental characteristics of late adolescence/young adulthood, ready access to the internet, and an expectation of computer/internet use. Majority of internet users in Nepal are aged 18–24 years, typical age group of undergraduate students [8]. So, this study helps to identify the prevalence of Internet addiction among undergraduate university students and to reveal factors associated with internet addiction in Kathmandu, Nepal.

## 2. Materials and Methods

### 2.1. Study Design, Period, Setting, and Population

This cross-sectional study was conducted among the undergraduate students of Kathmandu district of Nepal and data were collected during 30 June to 15 July of 2022.

### 2.2. Inclusion and Exclusion Criteria

Undergraduate students above the age of 18 years currently enrolled at any course of Pokhara University inside Kathmandu district were eligible to be included in this study and no exclusion were made in terms of any attributes.

### 2.3. Sample Size Determinations, Sampling Techniques, and Procedures

The past study conducted among higher secondary students in Kathmandu had noted the prevalence of internet addiction at 34.35% [16]. Considering this prevalence rate and margin of error at 5%, using Cochran's formula, the initial sample size was estimated at 312 which was optimized to 343 adjusting the 10% nonresponse rate.

Simple random sampling technique was used to choose six colleges from the Kathmandu district using a lottery method where a total of 344 students were enrolled at the time of the study. The required number of undergraduates from each selected colleges were estimated based on the number of students enrolled in each college and its programs to ensure proper representation of the undergraduate students. Finally, all the estimated number of students from each college and program were approached by enumerating all the students present in the randomly selected class at the time of data collection. When the number of students in class was more than the estimated sample size, the surplus data were also taken. The ethical approval for research was taken from IRC-CiST prior to the data collection and the approval was taken from concerned colleges to conduct the research. Informed consent was taken from the participants with explanation of aim and nature of the study prior to data collection.

### 2.4. Data Collection Tools and Procedures

Internet Addiction Test (IAT) is a validated instrument to measure Internet addiction [16]. The Internet Addiction Test was primarily developed by Dr. Kimberly Young, which is a 20-item 5-point Likert scale ranging from 0 to 5 (0 = less extreme behaviour to 5 = most extreme behaviour) that measures the severity of self-reported compulsive use of the internet [17]. The sum of the ratings was calculated for the 20-item responses for the total IAT score. The maximum IAT score is 100 points. IAT scores were categorized as internet users who scored <50 were considered average user and who scored ≥50 were considered internet addicted. The questionnaire consisted of four sections where the first section consisted of sociodemographic information, second section consist of behavioural factors related to the internet use, third section was about the perceived psychological status and interpersonal relationship, and the final section consisted of Young's 20-item Internet Addiction Test (IAT). The questionnaire was pretested among 10% of the sample population prior to data collection. Data were collected by the help of a self-administered method. During the process of data collection, the nature of the study was explained in detail to the participants; the details regarding the duration of the study, informed consent, and confidentiality concerns. Questionnaires were distributed to all the students possessing inclusion criteria.

### 2.5. Data Quality Control Issues

One of the researchers was self-involved for data collection and supervised by the other one. Each day at the end of data collection, every questionnaire was checked, reviewed by the supervisor, and organized for completeness and consistency. Pretesting of the questionnaire was carried out in one of the management colleges affiliated to Tribhuvan University in Kathmandu. Pretesting of the instrument was carried out in 10% of the sample. Cronbach's alpha of IAT tools was found to be 0.799 in this study.

### 2.6. Data Processing and Analysis

Data were entered in Epi-Data (version-3.1) and exported to Statistical Package on Social Sciences (IBM SPSS) version 25. The Pearson chi-squared test and binary logistic regression were applied to access the association between different independent variables and dependent variables (internet addiction) at 95% confidence interval and 5% level of significance i.e., *p* value<0.05. The unadjusted odds ratio (UOR) has also been reported along with the adjusted odds ratio (AOR) for those variables which were significant in bivariate analysis. For the multivariate analysis, the variance inflation factor (VIF) test was performed to check multicollinearity among the independent variables. The Hosmer–Lemeshow test (the HL test) for goodness-of-fit and Nagelkerke *R* square test were also performed for the model.

## 3. Results

A total of 400 questionnaires were distributed among the undergraduate students of the selected colleges, of which 344 questionnaires were received covering complete response to all the provided questions. Thus, the response rate of 86% was achieved.

### 3.1. Prevalence of Internet Addiction

Out of total 344 students who provided their complete response, 164 (47.7%) were found to had a mild level of Internet addiction under the IAT score of (31–49) whereas 96 (27.9%) were found to had a moderate addiction level (score 50–79) and 7(2.0%) were found to be severe addict (score 80–100). The overall prevalence of possible addict/internet addict based on IAT with a score of ≥50 was at 29.9% (95% CI: 25.0–34.9)

### 3.2. Sociodemographic Characteristics of Respondents

The mean age of the students who participated in the study was 20.68 ± 1.863 years while the minimum and maximum age of the participants ranged between 18 and 30 years. There was nearly equal participation of the female and male undergraduate students as 53.8% female and 46.2% were male. The majority were unmarried and reported to have a nuclear family (Table 1).

### 3.3. Internet Use-Related Characteristics of Respondents

With regards to the internet use pattern, most (208 i.e., 60.5%) of the participants reported have started using internet between 11 and 15 years of age. Likewise, more than half 201(58.4%) noted that they spent more than 5 hours per day over the internet. More than two-third (72.1%) of the students reported that their internet use is mostly for educational, recreational, and entertainment purposes, i.e., 256 (74.4%). Similarly, half of the participants (50.9%) reported that their social media as one of the major platforms for their internet use while 39 (11.3%) reported to be engaged in online jobs (Table 2).

### 3.4. Perceived Psychological Status and Interpersonal Relationship of Respondents

It was noted that nearly a quarter of the participants perceived themselves to be stressed and had a lower self-esteem. Almost two-third 226 (65.7%) reported having a wonderful relation with their parents while only few reported to have wonderful relation with their teachers and peers. More than half of the students i.e., 193 (56.1%) experienced loneliness sometimes in past one week (Table 3).

### 3.5. Factors Associated with Internet Addiction

In bivariate analysis, no statistically significant relationship was observed between student's sociodemographic characteristics such as age, gender, marital status, parental education, and economic status of the family and their internet addiction status (Table 4). In regards to the internet use pattern, a statistically significant relationship was observed between time spent by the students on daily basis over internet and their internet addiction status. Similarly, use of internet for social networking and media was also found to be associated with internet addiction at *p* < 0.05 (Table 5).

In context of perceived psychological factors and interpersonal relationship of the students, nature of students, relationship with their parents, parental control over their internet use, and the perceived level of loneliness were found to have a statistically significant relationship with their internet addiction status at *p* < 0.05 (Table 6).

For multivariate analysis, the variance inflation factor (VIF) test among the independent variables was performed where the highest reported VIF was 1.894 suggesting no issue of multicollinearity. Students who spent five hours or more on the internet were found to be almost twice more at odds (AOR: 1.780, 95% CI: 1.052–3.012) of experience internet addiction as compared to those who used internet for less than 5 hours a day. Similarly, higher odds of internet addiction were observed among the students who had good (AOR: 1.957, 95% CI: 1.022–3.745) and satisfactory (AOR: 2.832, 95% CI: 1.354–5.614) relation with their parents as compared to those who had wonderful relation. Likewise, students who reported their parents have higher control over their internet use were found to be thrice more at odds (AOR: 3.643, 95% CI: 1.687–7.863) of internet addiction as compared to students whose parents do not control their internet use. Students experiencing loneliness most of the time were also found to be have three-folds increase in their odds (AOR: 3.105, 95% CI: 1.264–7.629) of internet addiction in comparison to those who reported not experiencing loneliness (Table 7).

## 4. Discussion

It was seen that the prevalence of internet addiction among this study group was 29.90% which was supported by a meta-analysis conducted in prevalence of Internet addiction in medical students in different countries, the prevalence of IA was 30.1%, [18]. Furthermore, a study conducted among undergraduate university students in Ethiopia was found to be 29.4% moderate to severe IA [19]. Another study conducted among young adults in Bangladesh revealed prevalence of Internet addiction was 27.1%, [20], and online survey of problematic Internet use (PIU) and its correlates among undergraduate medical students of Nepal found the prevalence of PIU to be 31.9% [21].

A study conducted among medical students in Nepal found low prevalence of Internet addiction than this study where out of 100 students, 21 students were found to be slightly addicted to using the Internet [22]. In a study, among adolescents in a peri-urban setting in Nepal, 21.5% of the participants were identified with borderline Internet addiction and 13.3% with possible internet addiction [23]. A study among adolescent Turkish students prevalence of IA was 17.7%.

A study conducted among undergraduate students in Nepal revealed 35.4% prevalence of Internet addiction [24] and a study among higher secondary level students in Kathmandu district revealed possible addicts/Internet addicts to be 34.35% [16] which is slightly higher than this study.

The possible reason for variations in prevalence of Internet addiction across different country or even in the study conducted in same country might be because of sample size, sampling procedure, difference in social context and background of the participants, purpose of the Internet use, knowledge about the problems of Internet addiction, or aware about the proper use of Internet.

The association between time spent on Internet per day and IA was found to be statistically significant (*p* value 0.010). This association is supported by various studies such as a study conducted in Nepal [16], Ethiopia [19], Bangladesh [20], and Bengaluru, India [25]. As the dependence on Internet increases, individuals spent more time on Internet. Relationship with parents and IA have significant association (*p*  < 0.001) which is also indicated by several studies [16, 26–30]. Internet addiction was found to be higher among those who had satisfactory relationships within family. Self-control was negatively linked to Internet addiction as prevalence of Internet addiction was found to be higher in individuals with poor self-control than individuals with good self-control [30–32]. The loneliness of the participants was found to be significantly associated with Internet addiction (*p*  < 0.001) similar with the various studies [6, 33, 34]. Lonely individuals prefer to increase their communication through social networks to meet their emotional needs that was included in the meta-analysis conducted in Iran [35]. Perceived stress did not show any significant association with Internet addiction which is found to be consistent with the study conducted in East Malaysia [36] and the study conducted among Chinese adolescents [37]. However some previous studies reported significant association between perceived self-esteem and internet addiction, [38, 39] but we did not find the association i.e., low self-esteem increased susceptibility to IA. Therefore, more studies are needed to understand the significant association of self-esteem and IA.

Majority of the participants used internet for entertainment and refreshment purpose followed by education or to get new information. Use of Internet for social networking purpose showed significant association with IA (*p* value 0.024). This finding is lined with the study conducted in Northern Tanzania [40] and Saudi Arabia [41]. Most commonly used app/website was found to be social media among the participants supported by the study among young adults in Bangladesh [20].

The restrictive parenting approach was found to be significantly associated with IA (*p*  < 0.001) similar to the finding of the study conducted among adolescents in Hong Kong, the greater the number of rules and the stricter the enforcement of rules concerning the Internet use, the more likely it is that adolescents will become addictive users [29].

### 4.1. Strength and Limitations

The target population of this study was undergraduate university students of Nepal, which group is highly vulnerable to Internet addiction and majority of Internet users are also of this group. This study included a considerably high number of socio-demographic variables and also the Internet use and behavioural related and family and college related variables.

The finding of the study is based on the primary information collected using the standard tool by the active involvement of the researchers. Since this is a cross-sectional study conducted with minimal required sample size, it could not cover all disciplines and universities hence insufficient to conclude that Internet addiction is high among undergraduate students in Nepal. So, further studies using larger sample size is necessary for finding the causal associations on Internet addiction among undergraduate students in Nepal.

### 4.2. Implications of the Study

Problematic use of Internet/ Internet addiction can result in the various health problems and many factors are associated with it. This study identified the purpose of the Internet use, level of Internet addiction, and factors associated with Internet addiction. So, by identifying the factors associated with Internet addiction and strength of association between them, possible intervention action can be taken and will ultimately help to reduce the various health and educational problems, social life, and family relationship related problems arising due to excessive use of Internet.

## 5. Conclusions and Policy Recommendations

### 5.1. Conclusion

Prevalence of possible addiction of Internet use was about one-third among undergraduate students. There are numbers of factors which play a significant role to point out the alarming situation such as time spent on Internet per day, relationship with parents, parental control over Internet use, and loneliness feelings were found to be statistically associated with Internet addiction. Major purpose of Internet use was found to be entertainment and refreshment, and use of internet for social networking showed significant association with IA. Most commonly used app/website was found to be social media among the participants.

As family relationship and a restrictive parenting approach were found to be significantly associated, family-based prevention strategies need to be developed and applied to achieve healthy family interactions through improving parents-child communication and strengthening family functionality rather than directly restricting the Internet use. Further studies should be conducted in the consequences of problematic internet use in mental health.

### 5.2. Policy Recommendations

Family-based prevention strategies need to be developed and practice to achieve healthy family interactions through improving parents-child communication and strengthening family functionality rather than directly restricting the Internet use. Education programs need to be carried out on regular basis regarding the addictive behaviour and coping strategies also involving education and sensitization of the students as well as teachers.

## Figures and Tables

**Table 1:** Sociodemographic profile of the participants.

Characteristics	*n* (%)
*Age*	
<20 years	100 (29.1)
≥20 years	244 (70.9)

*Gender*	
Male	159 (46.2)
Female	185 (53.8)

*Marital status*	
Married	14 (4.1)
Unmarried	330 (95.9)

*Type of family*	
Nuclear	218 (63.4)
Joint/extended	126 (36.6)

*Education status of fathers*	
Illiterate	18 (5.2)
Literate	85 (24.7)
Primary level	47 (13.7)
Secondary level	83 (24.1)
Higher education	111 (32.3)

*Education status of mothers*	
Illiterate	35 (10.2)
Literate	79 (23.0)
Primary level	77 (22.4)
Secondary level	85 (24.7)
Higher education	68 (19.8)

*Family economic status*	
Low	17 (4.9)
Middle	310 (90.1)
High	17 (4.9)

**Table 2:** Internet use pattern.

Characteristics	*n* (%)
*Starting age of Internet use*	
<10 years	50 (14.5)
11–15 years	208 (60.5)
>16 years	86 (25.0)

*Time spent per day*	
<5 hours	143 (41.6)
≥5 hours	201 (58.4)

*Major use of Internet for education/recreation*	
Yes	248 (72.1)
No	96 (27.9)

*Major use of Internet for entertainment/refreshment*	
Yes	256 (74.4)
No	88 (25.6)

*Major use of Internet for Internet gaming*	
Yes	129 (37.5)
No	215 (62.5)

*Major use of Internet for social networking*	
Yes	175 (50.9)
No	169 (49.1)

*Major use of Internet for online jobs*	
Yes	39 (11.3)
No	305 (88.7)

*Major use of Internet to watch pornography*	
Yes	41 (11.9)
No	303 (88.1)

**Table 3:** Perceived psychological status and interpersonal relationship.

Characteristics	*n* (%)
*Perceived stress*	
Presence	101 (29.4)
Absence	243 (70.6)

*Perceived depression*	
Presence	17 (4.9)
Absence	327 (95.1)

*Perceived self-esteem*	
Low	101 (29.4)
High	243 (70.6)

*Relationship with parents*	
Wonderful	226 (65.7)
Good	65 (18.9)
Satisfactory	53 (15.4)

*Parental control over Internet use*	
Not at all	85 (24.7)
Sometimes	187 (54.4)
Often/almost always	72 (20.9)

*Relationship with teachers*	
Wonderful	108 (31.4)
Good	139 (40.4)
Satisfactory	97 (28.2)

*Relationship with peer*	
Wonderful	110 (32.0)
Good	154 (44.8)
Satisfactory	80 (23.3)

*Perceived level of self-control*	
Not at all	44 (12.8)
Sometimes	177 (51.5)
Often/almost always	123 (35.8)

*Perceived loneliness*	
Not at all	52 (15.1)
Sometimes	193 (56.1)
Often/almost always	99 (28.8)

**Table 4:** Internet addiction and its association with sociodemographic characteristics.

Variables	Internet addiction		*x* ^2^	*p* value
	Average user *n* (%)	Possible addict *n* (%)		
*Age*				
<20	77 (77.0)	23 (23.0)	3.239	0.072
≥20	164 (67.2)	80 (32.8)		

*Gender*				
Male	109 (68.6)	50 (31.4)	0.319	0.572
Female	132 (71.4)	53 (28.6)		

*Marital Status*				
Married	12 (85.7)	2 (14.3)	1.75	0.192
Unmarried	229 (69.4)	101 (30.6)		

*Type of family*				
Nuclear	146 (67.0)	72 (33.0)	2.701	0.100
Joint/extended	95 (75.4)	31 (24.6)		

*Fathers' education status*				
Higher education	112 (74.7)	38 (25.3)	2.693	0.101
Basic education	129 (66.5)	65 (33.5)		

*Mothers' education status*				
Higher education	135 (70.7)	56 (29.3)	0.079	0.778
Basic education	106 (69.3)	47 (30.7)		

*Family economic status*				
Low	13 (76.5)	4 (23.5)	3.280	0.194
Middle	213 (68.7)	97 (31.3)		
High	15 (88.2)	2 (11.8)		

**Table 5:** Internet addiction and its association with the Internet use pattern.

Variables	Internet addiction		*x* ^2^	*p* value
	Average user *n* (%)	Possible addict *n* (%)		
*Starting age of Internet use*				
<10 years	32 (64.0)	18 (36.0)	1.025	0.599
11–15 years	148 (71.2)	60 (28.8)		
>16 years	61 (70.9)	25 (29.1)		

*Time spent per day over Internet*				
<5 hours	111 (77.6)	32 (24.4)	6.676	0.010^*∗*^
≥5 hours	130 (62.2)	71 (35.3)		

*Major use of Internet for education/recreation*				
Yes	176 (71.0)	72 (29.0)	0.612	0.434
No	65 (67.7)	31 (32.3)		

*Major use of Internet for entertainment/refreshment*				
Yes	176 (68.8)	80 (31.3)	0.907	0.341
No	65 (73.9)	23 (26.1)		

*Major use of Internet for Internet gaming*				
Yes	86 (66.7)	43 (33.3)	1.132	0.287
No	155 (72.1)	60 (27.9)		

*Major use of Internet for social networking*				
Yes	128 (75.7)	41 (24.3)	5.112	0.024^*∗*^
No	113 (64.6)	62 (35.4)		

*Major use of Internet for online jobs*				
Yes	26 (66.7)	13 (33.3)	0.271	0.603
No	215 (70.5)	90 (29.5)		

*Major use of Internet to watch pornography*				
Yes	27 (65.9)	14 (34.1)	0.392	0.531
No	214 (70.6)	89 (29.4)		

^*∗*^Statistical significance at *p* < 0.05.

**Table 6:** Internet addiction and its association with the perceived psychological status and interpersonal relationship.

Variables	Internet addiction		*x* ^2^	*p* value
	Average user *n* (%)	Possible addict *n* (%)		
*Perceived stress*				
Presence	75 (74.2)	26 (25.8)	1.202	0.273
Absence	166 (68.3)	77 (31.7)		

*Perceived depression*				
Presence	9 (52.9)	8 (47.1)	2.498	0.114
Absence	232 (70.9)	95 (29.1)		

*Perceived self-esteem*				
Low	71 (70.3)	30 (29.7)	0.114	0.736
High	170 (70.0)	73 (30.0)		

*Relationship with parents*				
Wonderful	175 (77.4)	51 (22.6)	19.254	<0.001^*∗∗*^
Good	40 (61.5)	25 (38.5)		
Satisfactory	26 (49.1)	27 (50.9)		

*Parental control over Internet use*				
Not at all	69 (81.2)	16 (18.8)	15.487	<0.001^*∗∗*^
Sometimes	134 (71.7)	53 (28.3)		
Often/almost always	38 (52.8)	34 (47.2)		

*Relationship with teachers*				
Wonderful	82 (75.9)	26 (24.1)	5.950	0.054
Good	100 (71.9)	39 (28.1)		
Satisfactory	55 (59.1)	38 (39.2)		

*Relationship with peer*				
Wonderful	81 (73.6)	29 (26.4)	2.227	0.328
Good	109 (70.8)	45 (29.2)		
Satisfactory	51 (63.8)	29 (36.3)		

*Perceived level of self-control*				
Not at all	30 (68.2)	14 (31.8)	1.072	0.585
Sometimes	121 (68.4)	56 (31.6)		
Often/almost always	90 (73.2)	33 (26.8)		

*Perceived loneliness*				
Not at all	43 (82.7)	9 (17.3)	15.381	<0.001^*∗∗*^
Sometimes	143 (74.1)	50 (25.9)		
Often/almost always	55 (55.6)	44 (44.4)		

^*∗∗*^Statistical significance at *p* < 0.001.

**Table 7:** Predictors of Internet addiction.

Variables	Bivariate logistic regression		Multivariate logistic regression	
	UOR	95% CI	AOR	95% CI
*Time spent per day over Internet*				
<5 hours	Ref		Ref	
≥5 hours	1.894^*∗*^	1.163–3.087	1.780^*∗*^	1.052–3.012

*Major use of Internet for social networking*				
Yes	1.713^*∗*^	1.072–2.737	1.640	0.987–2.724
No	Ref		Ref	

*Relationship with parents*				
Wonderful	Ref		Ref	
Good	2.145^*∗*^	1.190–3.865	1.957^*∗*^	1.022–3.745
Satisfactory	3.563^*∗*^	1.912–6.639	2.832^*∗*^	1.354–5.614

*Parental control over Internet use*				
Not at all	Ref		Ref	
Sometimes	1.706	0.908–3.203	1.636	0.838–3.185
Often/almost always	3.859^*∗*^	1.889–7.880	3.643^*∗*^	1.687–7.863

*Perceived loneliness*				
Not at all	Ref			
Sometimes	1.671	0.760–3.671	1.667	0.715–3.886
Often/almost always	3.822^*∗*^	1.682–8.683	3.105^*∗*^	1.264–7.629

^*∗*^Statistical significance at *p* < 0.05, Nagelkerke *R* square: 0.185, Hosmer and Lemeshow chi-square: 5.015, *p*=0.756.

## Data Availability

The data used to support the findings of this study are available from the corresponding author upon request.

## Abbreviations

AOR: Adjusted odds ratio
CI: Confidence interval
HT test: Hosmer–Lemeshow test
IA: Internet addiction
IAI: Internet Addiction Index
IAT: Internet Addiction Test
IQR: Interquartile range
IRC-CiST: Institutional Review Committee CiST
PIU: Problematic Internet use
UOR: Unadjusted odds ratio
VIF: Variance inflation factor.
